# Activity of the integrase inhibitor S/GSK1349572 in subjects with HIV exhibiting raltegravir resistance: week 24 results of the VIKING study (ING112961)

**DOI:** 10.1186/1758-2652-13-S4-O51

**Published:** 2010-11-08

**Authors:** J Eron, JM Livrozet, P Morlat, A Lazzarin, C Katlama, T Hawkins, T Fujiwara, R Cuffe, C Vavro, J Santiago, M Ait-Khaled, S Min, JM Yeo

**Affiliations:** 1 UNC School of Medicine, Center for AIDS Research Clinical Care, NC, USA; 2 Hopital Edouard Herriot, Lyon, France; 3 Service de médecine interne et maladies infectieuses, Hôpital Saint André (CHU), Bordeaux, France; 4 San Raffaele Scientific Institute, Milan, Italy; 5 Hopital de la Pitie-Salpetriere, Paris, France; 6 Southwest CARE Center, Santa Fe, USA; 7 Shionogi & Co. Ltd., Osaka, Japan; 8 GlaxoSmithKline, Uxbridge, UK; 9 GlaxoSmithKline, Research Triangle Park, USA; 10 GlaxoSmithKline, Mississauga, Canada

## Background

S/GSK1349572(572) showed potent activity in Phase 2 studies in INI-naive HIV-infected subjects and limited cross-resistance to raltegravir (RAL) and elvitegravir in vitro. VIKING is an ongoing 24-week Phase 2b pilot study assessing 572 in subjects with RAL-resistant HIV. A good antiviral response during the functional monotherapy phase (through Day 11) of this pilot study was observed with a strong correlation between baseline susceptibility to 572 and response.

## Methods

27 RAL-experienced, adult subjects, with screening plasma HIV-1 RNA ≥1000c/mL and genotypic resistance to RAL and ≥ 2 other ART classes, received 572 50mg QD in Cohort I while continuing their failing regimen (without RAL). At Day 11 the background regimen was optimised, where feasible, and 572 continued. The antiviral activity (primary end-point at Day 11), tolerability, safety and virology data through Week 24 of Cohort I are presented. A higher dose is being assessed.

## Results

At Baseline, subjects harboured viruses displaying high level resistance to RAL (median fold change in susceptibility [FC] 161, range: 0.57- >166) and low median FC to 572 (1.46, range: 0.55-35). Median (IQR) Baseline CD4+ and plasma HIV-1 RNA were 110 cells/mm^3^ (40, 230) and 4.47 log10c/mL (3.9, 4.9), respectively. Median number (range) of prior ART drugs was 18 (10, 23). Twenty one (78%) subjects achieved plasma HIV-1 RNA<400 c/mL (n=11) or ≥ 0.7 log10 c/mL decline (n=10) at Day 11 (primary end-point). Post Day 11, the optimised background regimen (OBR) phenotypic susceptibility score (PSS) was 0, 1 and ≥ 2 for 12 (44%), 7 (26%) and 8 (30%) subjects, respectively. 17 subjects continued therapy through Week 24 when 14/27 (52%) and 11/27 (41%) subjects achieved < 400 c/mL and < 50 c/mL, respectively by TLOVR. Response correlated with OBR PSS: 2/12 (17%) subjects with PSS =0, 4/7 (57%) with PSS=1 and 8/8 (100%) with PSS ≥2 achieved <400 c/mL at Week 24. Drug related AEs (any grade) were observed in 6 (22%) subjects. Two subjects with advanced AIDS died after withdrawal from study for SAEs (brain mass, non-Hodgkin's lymphoma with febrile bone marrow aplasia) unrelated to 572.

## Conclusions

Despite high level baseline resistance to RAL and the limited activity of the OBR co-administered with 572, the majority of subjects achieved < 400 c/mL at Week 24 with improved response rates in those receiving at least one active background ART. S/GSK1349572 was generally well tolerated in this advanced population.

